# Supplementary material for: Feature-specific prediction errors and surprise across macaque fronto-striatal circuits
Source: Nat Commun. 2019 Jan 11;10:176. doi: 10.1038/s41467-018-08184-9 (PMC6329800; doi:10.1038/s41467-018-08184-9)
Supplement: Supplementary file 1 — Supplementary Information [file 41467_2018_8184_MOESM1_ESM.pdf]

Supplementary information

Feature-specific Prediction Errors and Surprise across Macaque Fronto-Striatal Circuits

Oemisch et. al.

Supplementary Methods

Anatomical reconstruction

Recording locations were identified using MRI images obtained following initial chamber placement. During MR scanning, we placed a grid marking the chamber center and peripheral positions as well as a diluted iodine solution inside the chamber for visualization. This allowed the referencing of target regions to the chamber center in the resulting MR images. Target regions (area 24 – ACC, area 46 – dlPFC, caudate nucleus, ventral striatum) were identified using the scheme from the Price lab[1,2]. The dorsal-ventral positioning of electrodes was estimated daily using the MRI images and audible profiles of spiking activity. The relative coarseness of the MRI images did not allow us to differentiate recording locations in the shell of the nucleus accumbens as opposed to the core of the nucleus accumbens with certainty.

Expectation maximization algorithm.

To identify at which trial during a block the monkey showed statistically reliable learning we analyzed the monkeys' trial-by-trial choice dynamics using the state–space framework introduced by[3], and implemented by[4]. This framework entails a state equation that describes the internal learning process as a hidden Markov or latent process and is updated with each trial. The learning state process estimates the probability of a correct (rewarded) choice in each trial and thus provides the learning curve of subjects. The algorithm estimates learning from the perspective of an ideal observer that takes into account all trial outcomes of subjects' choices in a block of trials to estimate the probability that the outcome in a single trial is correct or incorrect. This probability is then used to calculate the confidence range of observing a correct response. We defined the learning trial as the earliest trial in a block at which the lower

confidence bound of the probability for a correct response exceeded the p = 0.5 chance level. The identification of a learning trial allowed to discard blocks that were not learned.

Quantifying prediction errors with reinforcement learning modeling.

We quantified the trial-by-trial progression of RPEs during reversal performance using different computational model that were either based on reinforcement learning (RL) principles, on Bayesian tracking of reward probabilities, or combinations of those. The following describes details of the model similar to a previous study[5].

Feature-Nonselective RL model, F-NS model. The Feature-Nonselective RL model assigns values to features defining each stimulus. Each of the two stimuli are composed of three feature dimensions, the location (left (L) versus right (R)), the direction of motion (up (U) or down (D)) and the color (1 or 2). We label the six features with the indices 1 to 6, the corresponding values are thus denoted as V_i. A presented stimulus has a value for each of three feature dimensions, and thus possesses 3 feature value combinations (FVCs), the other stimulus has the remainder of the FVCs. All the FVCs of the chosen stimulus are updated, because each of them in principle could be a target that was rewarded, which of the three FVCs is the target can only be disambiguated across the presentation of multiple informative stimulus configurations. After receiving an outcome R (1/0 for rewarded/non rewarded) value updating is done according to

$$V_{i,t+1} = V_{\square,t} + \eta(R_t - V_{i,t}) \,, \qquad \text{(eq. 1)}$$

for all FVCs i that belong to the stimulus. This equation ensures that when there is a difference between the received reward and the expected (predicted) reward, the value gets updated to get

closer to the received reward. This implements the delta rule of classical prediction error learning, with η representing the learning rate.

The choice C_t of a stimulus is made by a softmax rule according to the sum of values of each FVC that belongs to the stimulus. We indicate the stimulus by the index j and the set of feature values that belong to it by s_j.

$$P(C_{t+1} = j) = \frac{\exp\left(\beta \sum_{i \in s_j} V_{i,t}\right)}{\sum_j \exp\left(\beta \sum_{i \in s_j} V_{i,t}\right)} \qquad \text{(eq. 2)}$$

Feature-selective RL model (F-S model). The Feature-Selective RL model uses the same mechanisms as the F-NS model, but selectively includes only the values and value-updates for the two colors (with no representation for the other dimensions). This model is inspired by a recent study[6] and assumes that the animals learning behavior would exclusively be driven by the color dimension without influences from non-color dimensions.

Feature-Decay RL model (F-Dec model). The F-Dec model is also an extension of the first F-NS model, and includes in addition a decay constant, which reduces the value of the FVCs of the stimuli that were not chosen. It is inspired by previous studies[5,7,8]. The feature values belonging to the chosen stimulus are updated according to eq. 1. The feature values i of the non-chosen stimulus decay according to

$$V_{i,t+1} = (1 - \omega) V_{i,t}\ , \qquad \text{(eq. 3)}$$

The decay parameter is denoted by ω. The choice is made as before (eq. 2).

Bayesian model. The remaining models are using a Bayesian mechanism to track the reward probabilities associated with each of the stimulus features. The learning goal of the Bayesian mechanism is to choose the stimulus that gives a reward, hence the one that has the target

feature (color 1 or 2). The information provided in each trial is accumulated across trials by using Bayes' rule, starting anew with a flat (zero) probability at the beginning of each block. This starts from the probability of obtaining a reward R_t as a function of the presented stimulus S_t and the choice C_t made assuming the feature value combination of the target stimulus is f: $p(R_t|C_t, f) = p_r R_t + (1 - p_r)(1 - R_t)$ and thus that the chosen stimulus S_{Ct} contains *f*. The expression tells us that the probability for getting reward ($R_t = 1$) is p_r and for getting no reward ($R_t = 0$) is $(1 - p_r)$. When the chosen stimulus S_{Ct} does not contain *f*, $p(R_t|C_t, f) = p_n R_t + (1 - p_n)(1 - R_t)$. We can combine these two expressions into one by defining $S_{Ct}(f) = 1$, when it contains feature *f*, and zero otherwise yielding

$$p(R_t|C_t, f) = S_{C_t}(f)[p_r R_t + (1 - p_r)(1 - R_t)] + (1 - S_{C_t}(f))[p_n R_t + (1 - p_n)(1 - R_t)]. \quad \text{(eq. 4)}$$

The calculations simplify further when choosing $p_n = 1 - p_r$. What we are interested in is $p(f|\mathcal{D}_{1:t})$, and aim to express it iteratively in terms of $p(f|\mathcal{D}_{1:t-1})$. We start the iteration from a uniform initial distribution in each block, reflecting the lack of knowledge about the target. Each trial gives independent information, hence we can write

$$p(f|\mathcal{D}_{1:t}) = p(f|R_t, C_t)p(f|\mathcal{D}_{1:t-1}) = \frac{p(R_t|C_t, f)p(f)}{p(R_t)} p(f|\mathcal{D}_{1:t-1}) \quad \text{(eq. 5).}$$

The expression depends only on f and factors that do not depend on f, such as $p(R_t)$, will be taken into account as a consequence of normalization of this probability distribution across *f*. On trial t, when ignoring the past, target f could be anything, hence $p(f)$ is constant, we thus obtain:

$$p(f|\mathcal{D}_{1:t}) \propto p(R_t|C_t, f)p(f|\mathcal{D}_{1:t-1}), \quad \text{(eq. 6)}$$

where after each update we need to normalize this distribution again. The model uses as a 'value' the probability of reward on a new trial, as a function of the choice (still to be made), given the past data:

$$V_{i,t} = p(R_{t+1}|C_{t+1}, \mathcal{D}_{1:t}) = \sum_f p(R_{t+1}|C_{t+1} = i, f)p(f|\mathcal{D}_{1:t}) \qquad \text{(eq. 7)}$$

The choice is made in the same way as before using a Boltzman function with parameter β:

$$P(C_{t+1} = i) = \frac{\exp(\beta V_{i,t})}{\sum_j \exp(\beta V_{j,t})} \qquad \text{(eq. 8)}$$

Feature-Dimension weighted RL model (F-DW model). The F-DW model combines Bayesian weighting of reward probabilities for different feature dimensions with RL mechanisms. This model was introduced before to account for behavioral adjustments of choices in a multidimensional visual learning task and we recently validated it as a model accounting for feature-based reversal learning in the macaque[5]. The model represents the stimuli in terms of their stimulus dimension (color, motion, location), features (color A, color B, downward motion, upward motion, left, right), and the actual combinations of features for stimulus 1 and stimulus 2.

The model uses Bayesian inference about which stimulus feature dimension f (color, motion or location) is the likely target dimension via $p(f|\mathcal{D}_{1:t})$ to obtain a dimension-weighted representation for each stimulus. For tracking target feature probability, we denote the feature dimension as f_d (1: location, 2: direction of motion, 3: color) and for each d, f_d, takes two values 1 and 2. For instance, f_3=1 indicates the first color. We then calculate the probability for the rewarded stimulus (the target) to have dimension d, $p_d = p(d|\mathcal{D}_{1:t}) = \sum_{f_d=1,2} p(f_d|\mathcal{D}_{1:t})$. This defines a feature dimension weight $\phi_d = \frac{p_d^\alpha}{\sum_{d'} p_{d'}^\alpha}$, with exponent α and normalized to yield

a sum across dimensions equal to one. The predicted reward value of a feature is then denoted by W_{f_d} and scaled by the dimensional weight ϕ_d.

The value of the specific stimulus i is given by the sum across all weighted feature values that are part of the stimulus

$$V_i = \sum_d \phi_d W_{f_d} \qquad \text{(eq. 9)}$$

The choice of which stimulus is selected on a given trial is implemented with a softmax rule using the Boltzmann function as in equation 8.

Following a choice, the stimulus values of the chosen stimulus are updated by a reward prediction error, RPE = $(R_t - W_{i,t})$, scaled by learning rate η according to:

$$W_{f_d,t+1} = W_{f_d,t} + \eta(R_t - W_{i,t}) \qquad \text{(eq. 10)}$$

Positive RPEs ranged in value from 0 to 1, negative RPEs ranged in value from -1 to 0.

In summary, the *F-DW model* updates feature values of the chosen stimulus using the RPE $(R_t - W_{i,t})$ and separately scales feature values by a dimensional weight calculated using Bayesian updating of how the dimensions color, motion and location relate to reward outcomes.

Feature-Dimension weighted-Decay RL model (F-DW-Dec model). The Feature-Dimension weighted-Decay RL model (F-DW-Dec model) is using the same architecture as the F-DW- model with the addition of a decay parameter for features of the nonchosen stimulus. Feature values of the unchosen stimulus were scaled down (decayed) by $(1 - \omega)$, similar to previous studies[5,7] according to:

$$W_{f_d,t+1} = (1 - \omega)W_{f_d,t} \qquad \text{(eq. 11)}$$

Procedures for optimization, evaluation and comparison of the different models are described in the Methods section.

Cell-type specificity of RPE encoding neurons.

For the set of highly isolated neurons (monkey H: n = 428, monkey K: n = 398), we aligned, normalized, and averaged all action potentials[9]. To distinguish putative interneurons (narrow-spiking) and putative pyramidal cells (broad-spiking) in dlPFC and ACC, we analyzed the peak-to-trough duration and the time for repolarization for each neuron[10]. The time for repolarization was defined as the time at which the waveform amplitude decayed 15% from its peak value. We computed the principal component analysis (PCA) and used the first component because it allowed for better discrimination between narrow- and broad-spiking cells, compared to any of the two measures alone (Hartigan dip test, $p < 0.0005$). In addition, a comparison of Akaike's and Bayesian was used to confirm that a two-Gaussian model fit the data better than a one-Gaussian model. To distinguish putative none-spiny interneurons from putative medium-spiny neurons (MSNs) in CD and VS, we analyzed the peak width (at half maximum) and Initial Slope of Valley Decay (ISVD)[11,12], as they provided a better waveform discrimination than e.g. peak-to-trough duration. The ISVD was computed as follows:

$$ISVD = 100 * \frac{(V_T - V_{0.26})}{A_{PT}} \qquad \text{(eq. 12)}$$

where V_T is the most negative value (trough) of the spike waveform, $V_{0.26}$ is the voltage at 0.26 ms after V_T, and A_{PT} is the peak-to-trough amplitude[11]. Although we could not discard unimodality for the first PCA component (or for either of the single measures, Hartigan dip test, $p > 0.05$), Akaike's and Bayesian information criteria confirmed that a two-Gaussian model fit the data better than a one-Gaussian model. For frontal and cingulate units, we then

used the two-Gaussian model and divided neurons into two groups of narrow and broad spiking units. For striatal units, because we could not discard unimodality for the first PCA component, we used the two-Gaussian model and defined two cutoffs that divided neurons into three groups. The first cutoff was defined as the point at which the likelihood of a narrow-spiking/putative interneuron was 3 times larger than the likelihood of a broad-spiking cell, and vice versa for the second cutoff. We reliably classified dlPFC/ACC neurons (n = 485) as either putative pyramidal cells (broad spiking, n = 344, monkey H: 203, monkey K: 183) or putative interneurons (narrow-spiking, n = 141, monkey H: 78, monkey K: 49). Therefore, in monkey H 72% of neurons in ACC/dlPFC were identified as putative pyramidal cells while 28% of neurons were identified as putative interneurons. In monkey K, 82% of neurons were identified as putative pyramidal cells and 17% as putative interneurons. We classified 96% of striatal neurons (n = 277) as either putative MSNs (broad spiking, n = 198, monkey H: 96, monkey K: 113) or putative none-spiny interneurons (narrow-spiking, n = 79, monkey H: 35, monkey K: 36), while n = 26 (monkey H: 8, monkey K: 11) neurons fell in between the criteria and could not be reliably classified. Therefore, in monkey H 73% of neurons in CD/VS were identified as putative MSNs while 27% of neurons were identified as putative interneurons. In monkey K, 77% of neurons were identified as putative MSNs and 23% as putative interneurons. For striatal units, we additionally verified our classification by comparing the firing rates between neurons classified as MSNs and those classified as interneurons. Striatal interneurons tend to be fast-spiking interneurons and should have a higher firing rate than the relatively low-firing MSNs [12,13]. Indeed, in both monkeys, neurons classified as interneurons had a higher mean firing rate (monkey H: 4.96±1.1 Hz, monkey K: 4.77 ± 2.62 Hz) than neurons classified as MSNs (monkey H: 1.70 ± 0.38 Hz, monkey K: 1.61 ± 0.26 Hz), and this was statistically reliable in both monkeys (t-test, monkey H: $p < 0.001$, monkey K: $p = 0.039$). For the analysis of narrow versus broad spiking feature-specific versus non-specific RPE units we combined

data from both monkeys because of relatively low neuron numbers. Proportions of narrow versus broad spiking units between non-specific and feature-specific RPE neurons were compared using chi-square statistics.

Stimulus selection following low and high prediction errors.

We tested how neurons that encoded a color-specific prediction error changed their firing rate during color selection in trials following low versus high prediction errors (Figure 9 main text). To do so, we identified for each color-specific RPE neuron the 25% of trials with the greatest prediction errors (from the model) and those 25% with the lowest prediction errors. These trials were then split into whether the choice was made to the color for which an RPE was encoded (preferred color) and those for which a choice was made to the other color (non-preferred color). For each trial n we found trial n+1 and computed the normalized change in firing rate from the 400-0ms prior to stimulus color onset to the 100-700ms following stimulus color onset according to the following:

$$\Delta FR_n = \frac{mFR_{post-col} - mFR_{pre-col}}{mFR_{post-col} - mFR_{pre-col}} \quad \text{(eq. 13)}$$

where ΔFR_n is the normalized change in firing rate that ranges from -1 to 1. We thus computed for each neuron the average rate change (1) following low RPE trials in which the preferred RPE color (i.e. the color associated with a higher RPE) was chosen, (2) following low RPE trials in which the non-preferred RPE color was chosen, (3) following high RPE trials in which the preferred RPE color was chosen, and (4) following high RPE trials in which the non-preferred RPE color was chosen. Across the populations of color-specific positive RPE, negative RPE, or unsigned RPE encoding neurons, we then compared the change in firing rate at stimulus selection following high versus low RPE trials for the preferred color and for the non-preferred color choice trials using paired t-tests. In a second step, we split neuron

populations based on their respective recording locations and performed the equivalent analysis.

Task variables encoded in the outcome epoch.

To characterize neural responses in the outcome epoch, we adapted an analysis procedure from Padoa-Schioppa and colleagues[14–16] (http://accl.psy.vanderbilt.edu/resources/code/). We tested whether neurons encoded any of twelve variables at the time of reward onset/omission. These twelve variables included the three stimulus features (color, location, motion) i) selected in the current choice independent of choice outcome (correct and error) (chosen color, chosen location, chosen motion)[17], ii) selected in the previous choice (trial n-1) independent of choice outcome (correct and error) (previous chosen color, previous chosen location, previous chosen motion)[17,18], iii) of the target independent of choice (correct and error) (target color, target location, target motion)[19], in addition to the variables outcome (correct and error), previous outcome (correct and error)[18] and learning progress (correct trials during learning versus after learning as obtained from the EM algorithm described above). To estimate the correlation between the twelve variables, we computed the correlation coefficient between any two trial vectors of the variables per recording session and then computed the average absolute correlation coefficient across recording sessions (the average correlation coefficient now varies between 0 and 1). The correlation matrix is shown in Supplementary Figure 2C. To identify whether any neuron encoded any one variable, we performed independent linear regressions for each neuron on each variable. A neuron's firing rate was averaged in the 0.1 - 0.7 seconds after reward onset/omission and was considered to significantly encode a variable at $p \leq 0.05$. In general, a neuron's response could be explained by multiple variables, which is likely because variables are correlated with each other, a situation referred to as multi-collinearity. We therefore adapted the "best-subset" method as a method of variable selection used in the

case of multi-linear regressions[16,20,21].

Best-subset method. We computed for each subset of d variables the total number of neural responses explained by that subset and determined which subset explained the maximum number of responses. This was repeated for d=1, 2, 3... variables per subset. We determined the number of variables necessary to characterize the population when 85% of the maximum number of responses explained was reached. The best-subset method assumes that each neuron only encodes a single variable. We therefore tested for second-order encoding to determine the proportion of neurons that encoded more than one variable[16].

Second order encoding. We found for each neuron the best-fit variable and its corresponding R^2 value. To determine whether adding an additional variable to the regression led to a significantly higher R^2 value, we computed:

$$F_{X,Y} = \frac{(n-3)*(R^2_{XY} - R^2_X)}{(1-R^2_{XY})} \quad \text{(eq. 14)}$$

where R^2_X is from the original linear regression on X only, R^2_{XY} is from the bilinear regression on X and Y and n is the number of trials. $F_{X,Y}$ is computed for each of the eleven possible second variables and the maximum F is found. If the corresponding p-value for the maximum F value is ≤ 0.01, we consider the neuron to significantly encode the second variable[14–16]. 31% of neurons significantly encoded a second task variable, which is more than expected by chance (binomial test, $p < .0001$). The major variables that were multiplexed at the second order were previous trial outcome (17.8%) and learning progress (26.7%), with both of these more often encoded at the second order than expected based on an equal distribution across all twelve variables (binomial test, $p < .001$).

Supplementary Figure 1

a

Model Names and abbreviation		Akaike-Information Criterion	
		Monkey H	Monkey K
Feature-Nonselective RL	F-NS	49436	23177
Feature-Selective RL	F-S	44558	21001
Feature-Dimension Weighted RL	F-DW	44905	20744
Feature-Decay RL	F-Dec	46910	22222
Feature-Dimension Weighted-Decay	F-DW-Dec	44268	20701

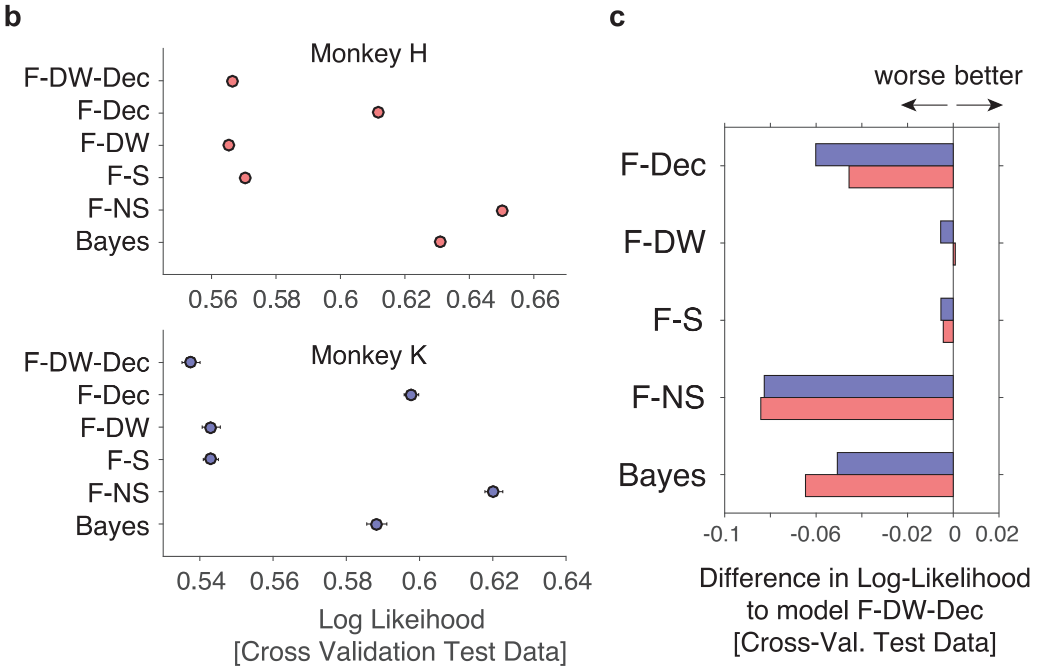

Supplementary Figure 1 | Evaluation of reinforcement learning model performance.

(a) The Akaike Information Criterion (AIC) for five RL models optimized to fit the learning performance of each monkey. Lower AIC values indicate a better model fit after penalizing for the number of free parameters (k) used in the models. AIC is calculated as [2k - 2ln(Log-Likelihood)]. The two numbers with shaded background indicate that the Feature-Dimension Weighted Decay RL model has the best AIC for each monkey. (b) Log-Likelihood results for the cross-validation test data for five models. For both monkeys (H and K in upper and lower panel, respectively) the three models with the best cross-validation performance (lowest Log-

Likelihoods) have mechanisms that focuses the value learning to specific feature-dimensions (Feature-Selective RL, Feature-Dimension Weighted RL, and Feature-Dimension Weighted Decay RL). (c) The difference in Log-Likelihood for all models relative to the Feature-Dimension Weighted Decay RL that showed the best AIC score for both monkeys (see a). Lower values indicate worse Log-Likelihoods.

Supplementary Figure 2

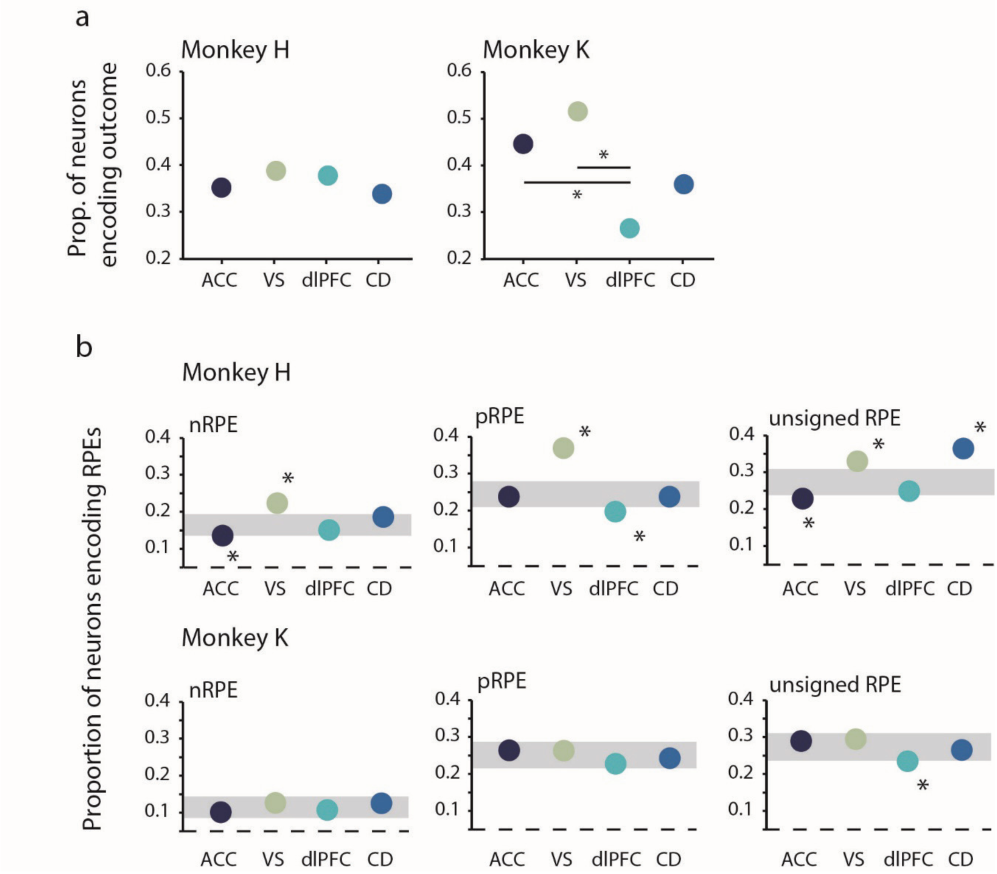

Supplementary Figure 2 | Outcome and RPE encoding.

(a) Shown are the proportion of neurons in each area that significantly encoded whether the current trial outcome was correct or incorrect within 0.1 – 0.7 seconds following feedback onset for monkey H (right) and monkey K (left) (Regression analysis, $p \leq 0.05$). An asterisk indicates a significant difference in proportions (Chi-square test, Bonferroni-Holm multiple comparison corrected, $p \leq 0.05$). (b) Proportion of neurons encoding negative, positive and unsigned RPEs. Shown are the proportion of neurons in each area that significantly correlate their firing rate with a negative prediction error on error trials (top), with a positive prediction error on correct trials (middle), or with an unsigned prediction error (bottom) for monkey H (left) and monkey K (right). Grey bars represent upper and lower bootstrap confidence intervals. An asterisk indicates $p < .05$ by falling outside of the specified confidence interval.

Supplementary Figure 3

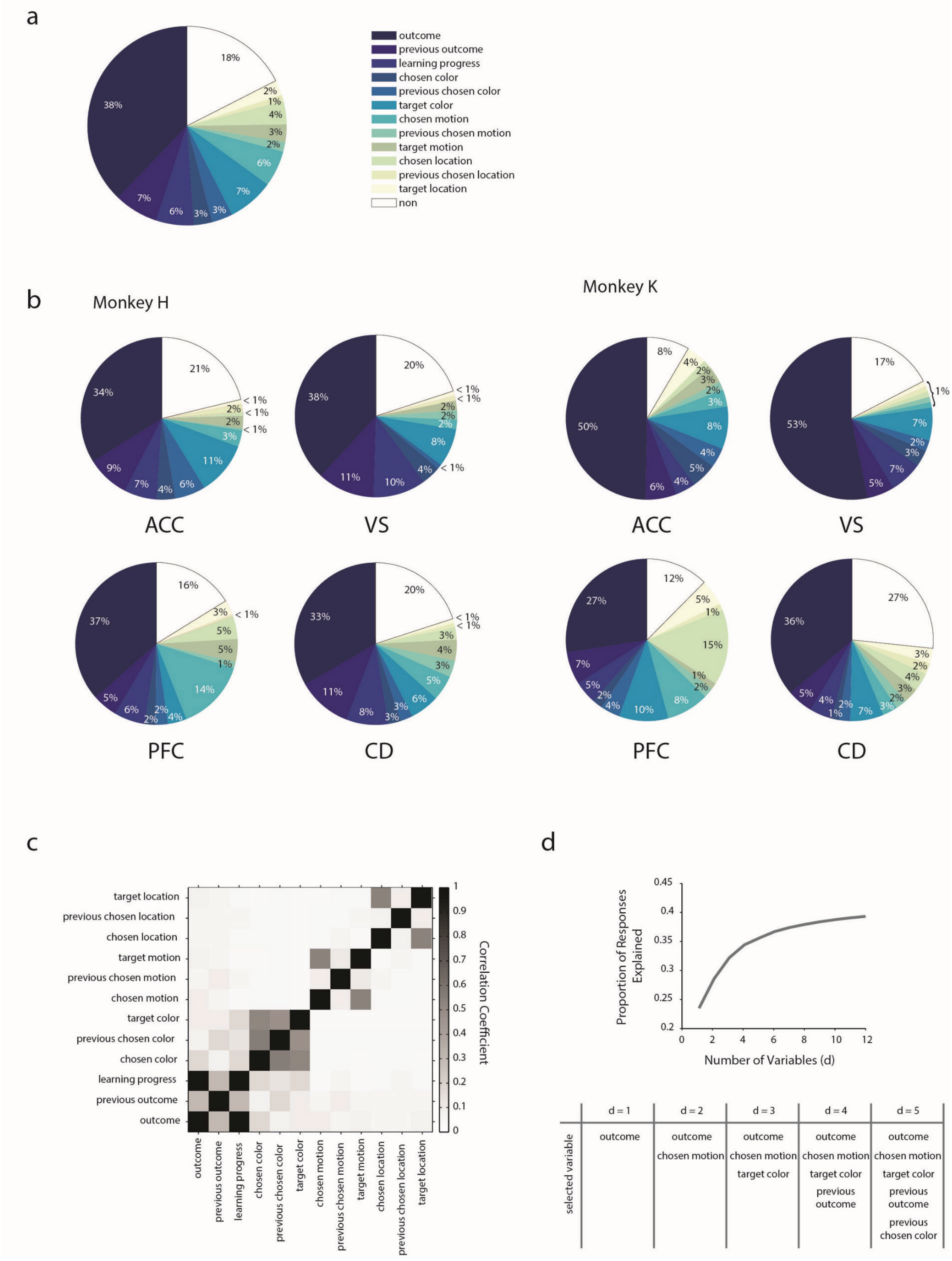

Supplementary Figure 3 | Task variables encoded in the outcome epoch.

We determined for each neuron whether it encoded any of twelve task variables in the outcome epoch (0.1 – 0.7 sec.) using single linear regressions and initially assuming each neuron encoded only a single variable (first order)[15,16]. (a) Proportion of variables encoded in the outcome epoch across areas and monkeys. Neurons are grouped based on the variable that provided the best fit for their responses (highest R^2, linear regression). (b) Conventions are as in (A), but separately for areas and monkeys. (c) Correlation matrix for all twelve variables tested. Elements of the correlation matrix vary between 0 and 1. Note that the variables outcome and learning progress are shown with a correlation of 1, because the variable learning progress contained correct trials only. (d) Proportion of responses explained for each added variable based on the best-subset selection procedure (see Supplementary Methods). The maximum percentage of responses that could be explained using all twelve variables was 39.3%, whereby 85% of this maximum number of responses could be explained with four variables. The best-subset method relies on the assumption that each neuron encodes a single variable only. The previous therefore suggest that a substantial proportion of neurons encoded more than just one variable. Bottom: Best variable subsets for the first five variable combinations that explain the largest number of responses. The fact that the same sets of variables are included in best subset sizes 2, 3, 4 and 5 indicates the robustness of these results.

Supplementary Figure 4

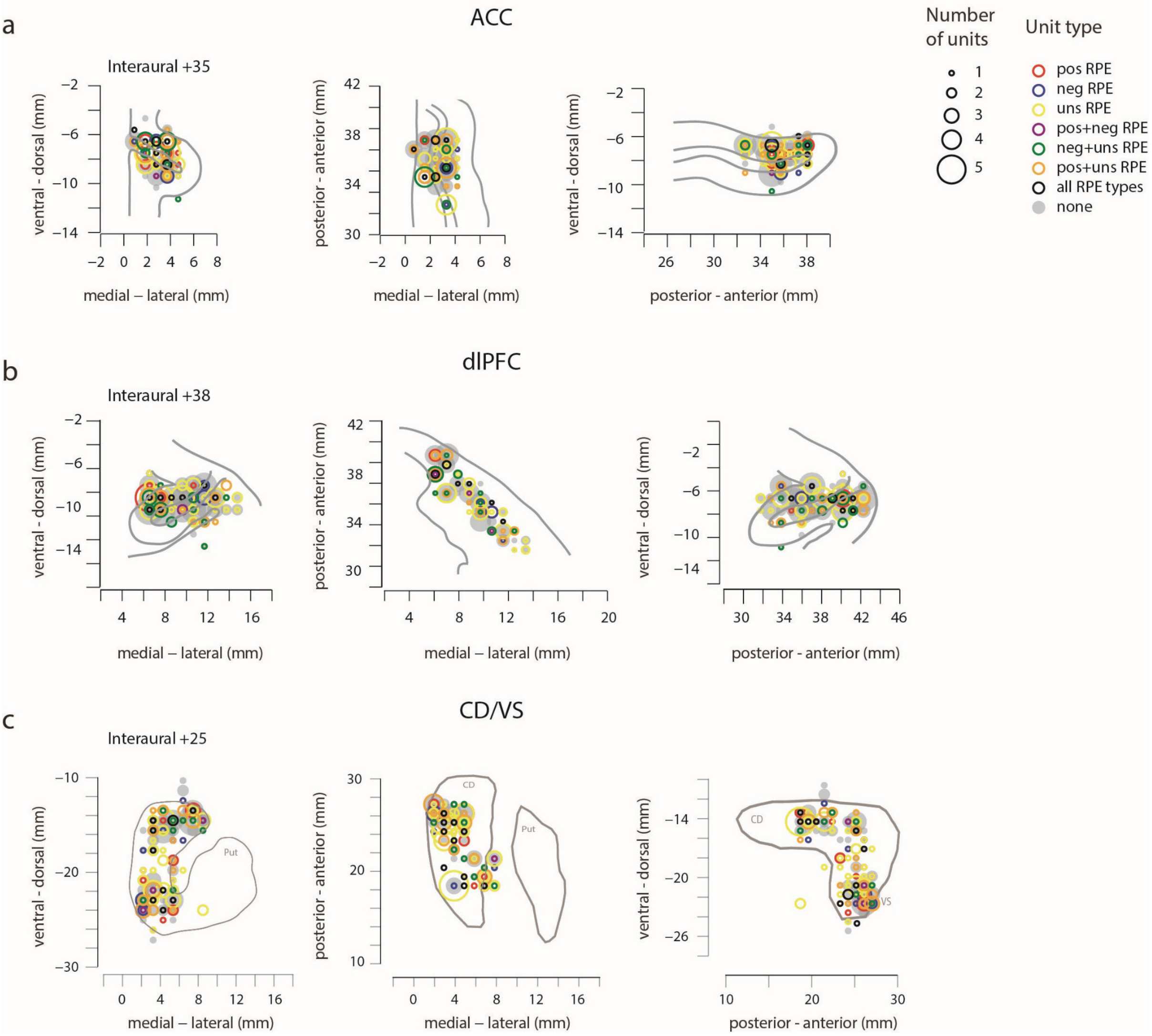

Supplementary Figure 4 | Anatomical locations of RPE neurons in monkey H.

Feature-specific RPE encoding neurons are differentiated with colors based on the RPE type they encoded (positive RPE (pos), negative RPE (neg), unsigned RPE (uns)) and whether they encoded more than one RPE type. Circle size refers to the number of neurons represented at that x-y location. Displayed are three orientations: coronal view (top left), axial view (right), and sagittal view (bottom left) for ACC (a), dlPFC (b) and Striatum (c). Within a given orientation, units are collapsed across the third non-visible orientation. Coordinates are relative to stereo-tactic zero.

Supplementary Figure 5

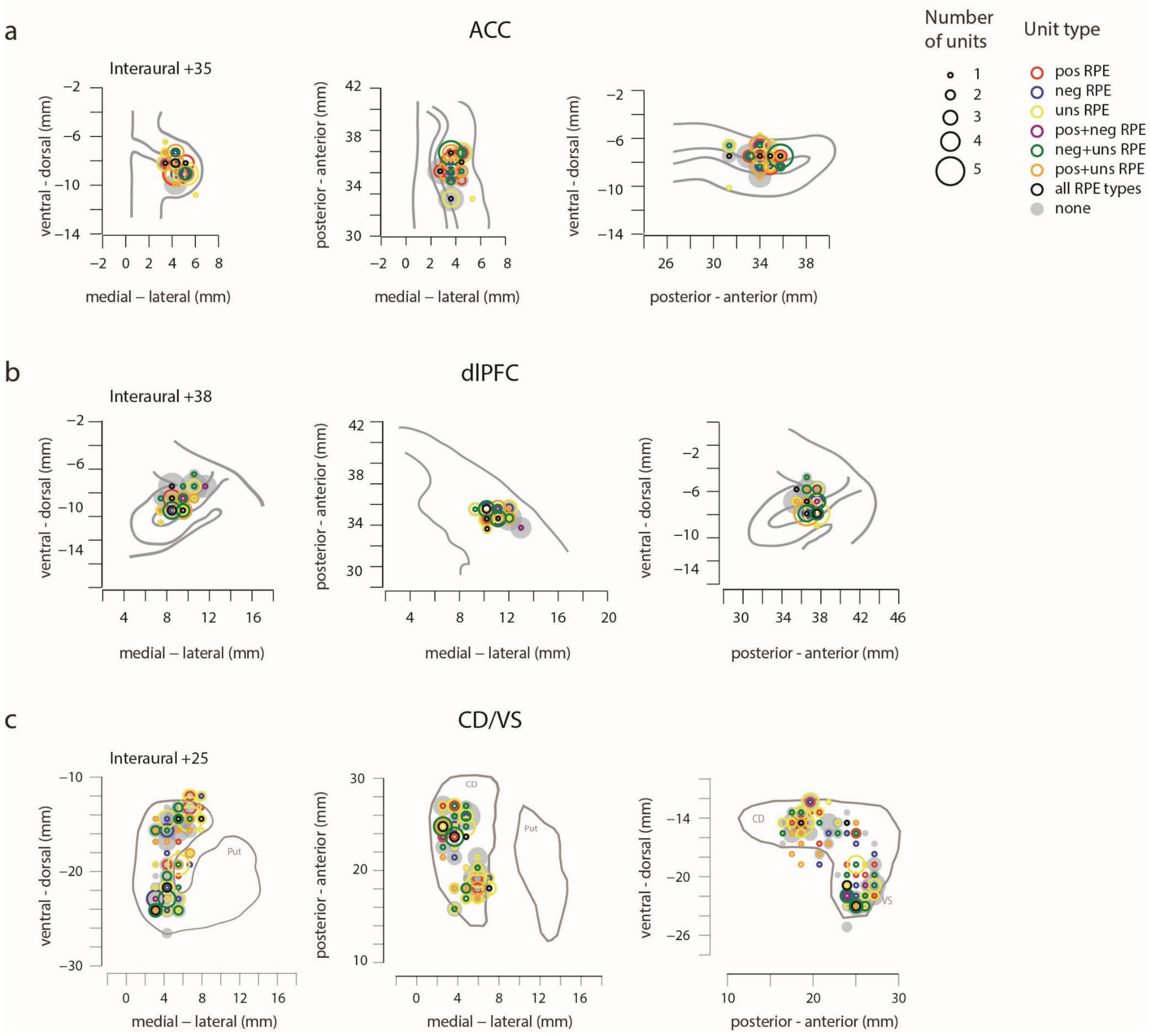

Supplementary Figure 5 | Anatomical locations of RPE neurons in monkey K.

Same conventions as Supplementary Figure 4.

Supplementary Figure 6

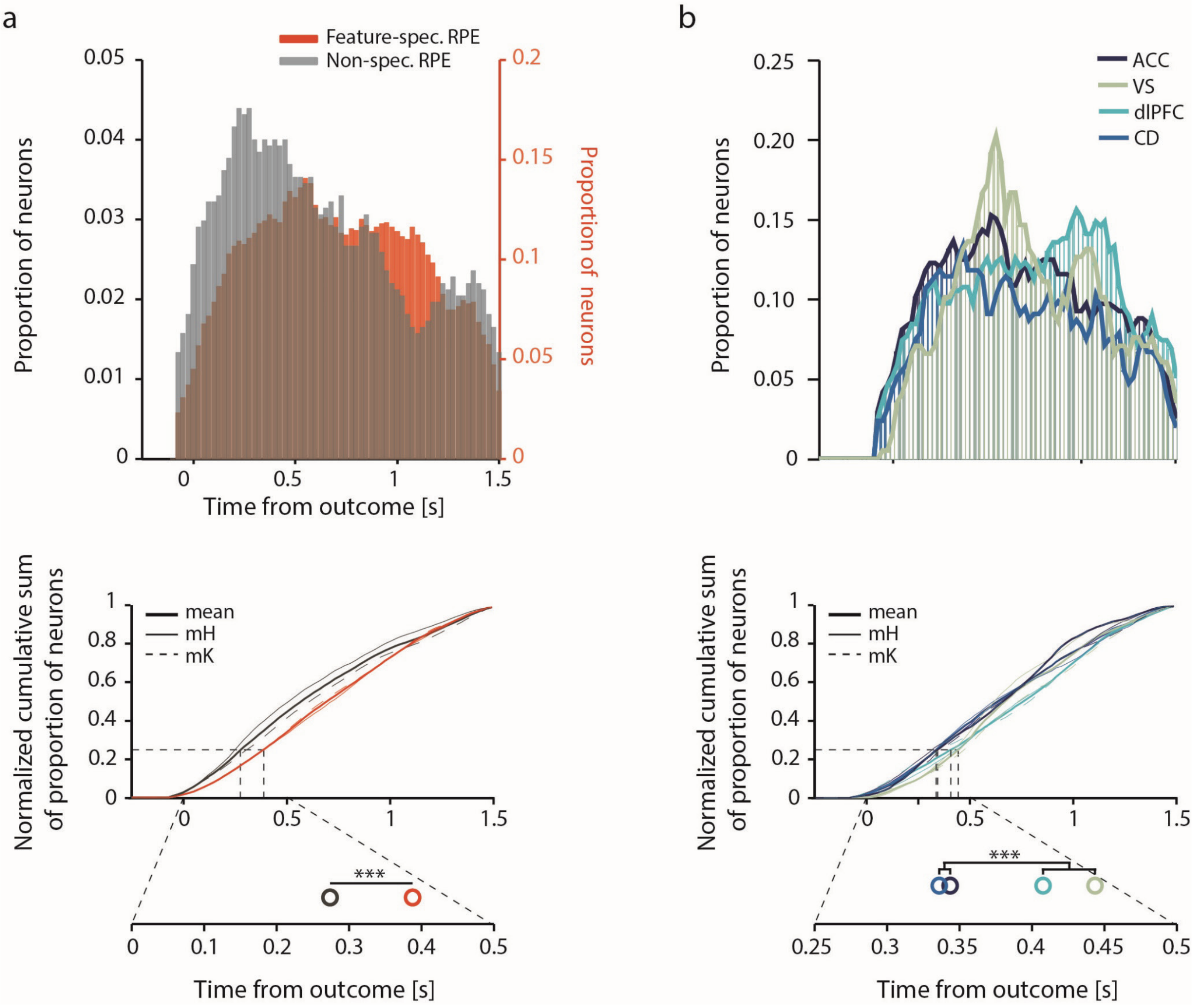

Supplementary Figure 6 | Temporal profile of feature-specific and non-specific RPE.

For this analysis, we increased the threshold for identifying RPE encoding units from four consecutive bins to six consecutive bins. (a) Top: Histogram of the proportion of units encoding feature-specific RPEs and non-specific RPEs in time combined across both monkeys. For each neuron, all time bins for which an RPE was encoded are included ($n_{feat\text{-}spec} = 545$; $n_{non\text{-}spec} = 204$). These histograms differed significantly from each other (Kolmogorov-Smirnoff test, Bonferroni-Holm corrected: $p_{feat\text{-}non} < .001$; Rank sum test, Bonferroni-Holm corrected: $p_{feat\text{-}non} = .006$) Bottom: Normalized cumulative sums of the histograms above. Thick lines represent

the mean across both monkeys, thin continuous (dotted) lines represent cumulative sums of monkey H (K). Cumulative sum of feature-specific and non-specific RPEs differed significantly (Kolmogorov-Smirnoff test, Bonferroni-Holm corrected; both $p < .001$). Shown below is the magnification - open circles represent time points at which 25% of respective signal was encoded (non-specific: 260ms, feature-specific: 370ms). Three asterisks indicate $p < .001$ (randomization procedure). Conventions are equivalent to Figure 3 of the main text. (b) Top: Histogram of the proportion of feature-specific RPE encoding units in ACC, VS, dlPFC and CD combined in time across both monkeys. For each neuron, all time bins for which an RPE was encoded were included ($n_{ACC} = 197$; $n_{VS} = 87$; $n_{dlPFC} = 167$; $n_{CD} = 94$). Bottom: Normalized cumulative sums of the histograms above. Cumulative sums of all areas except for ACC and CD differed significantly from each other (Kolmogorov-Smirnoff test, Bonferroni-Holm corrected; $p_{ACC\text{-}CD} = .94$, all other $p < .001$). Shown below the magnification - open circles represent time points at which 25% of feature-specific RPEs is encoded in the four areas (ACC: 335ms, VS: 440ms, PFC: 403ms, CD: 333ms). Three asterisks indicate $p < .001$ (randomization procedure). All conventions are equivalent to Figure 4 of the main text.

Supplementary Figure 7

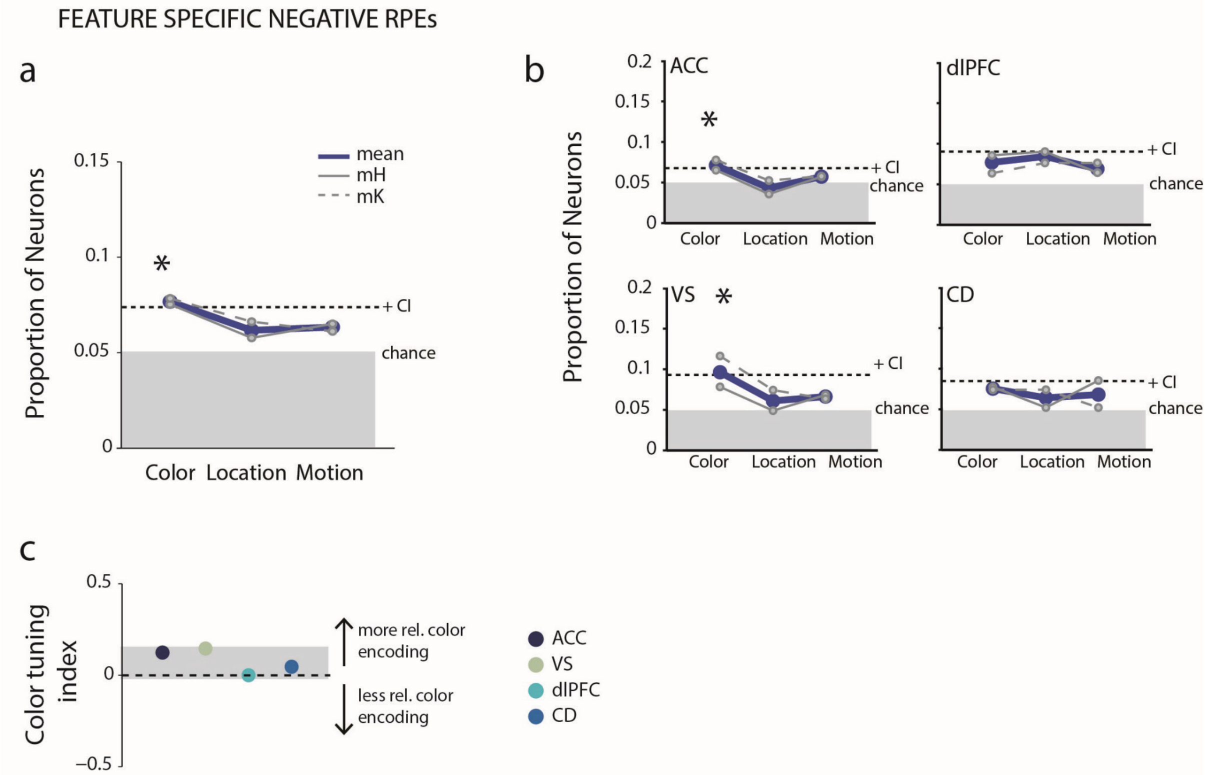

Supplementary Figure 7 | Prevalence of feature-specific negative RPE encoding.

For this analysis, negative RPE encoding neurons were alternatively identified as those neurons that significantly decreased their firing rates with more negative RPE values. Shown are proportions of neurons that encode a color-, location-, or motion-specific negative RPE signal either combined across areas (a) or split by areas (b). Thick blue lines represent averages across both monkeys. Thin continuous grey lines represent data from monkey H, thin dashed grey lines represent data from monkey K. An asterisk indicates $p \leq .05$ using a one-sided bootstrap procedure that randomized the feature labels. Dotted lines indicate upper confidence interval. Grey bars indicate chance level proportion at 0.05. (c) Color tuning indices for each area computed according to eq. 2. Grey bar represents upper and lower bootstrap confidence interval. Color tuning indices did not differ between areas.

Supplementary Figure 8

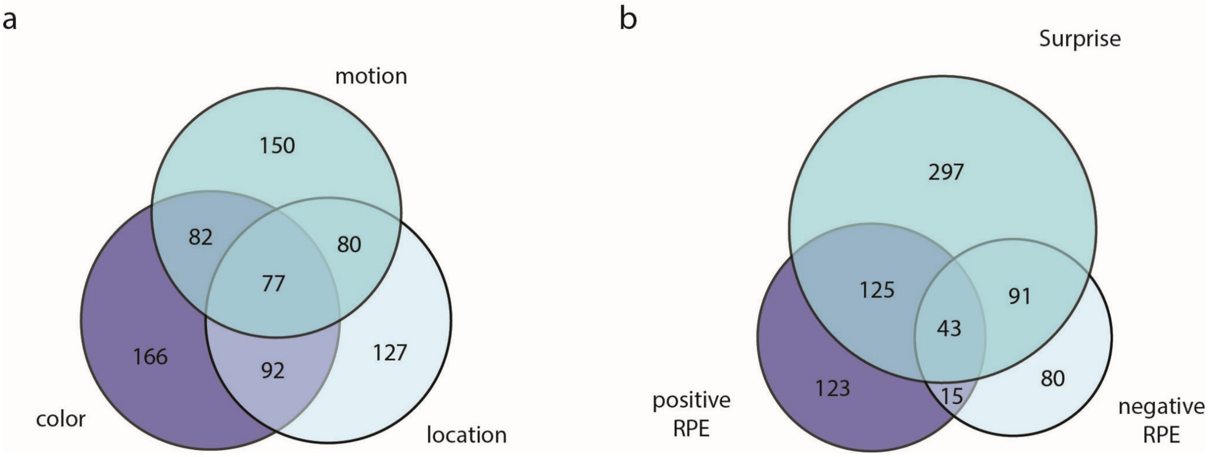

Supplementary Figure 8 | Overlap of neurons encoding feature-specific RPEs.

Overlap in neural populations encoding the different feature-specific RPEs split by object dimension (a) and RPE type based on trial outcome (b). Numbers inside circles refer to numbers of neurons within that category.

Supplementary Figure 9

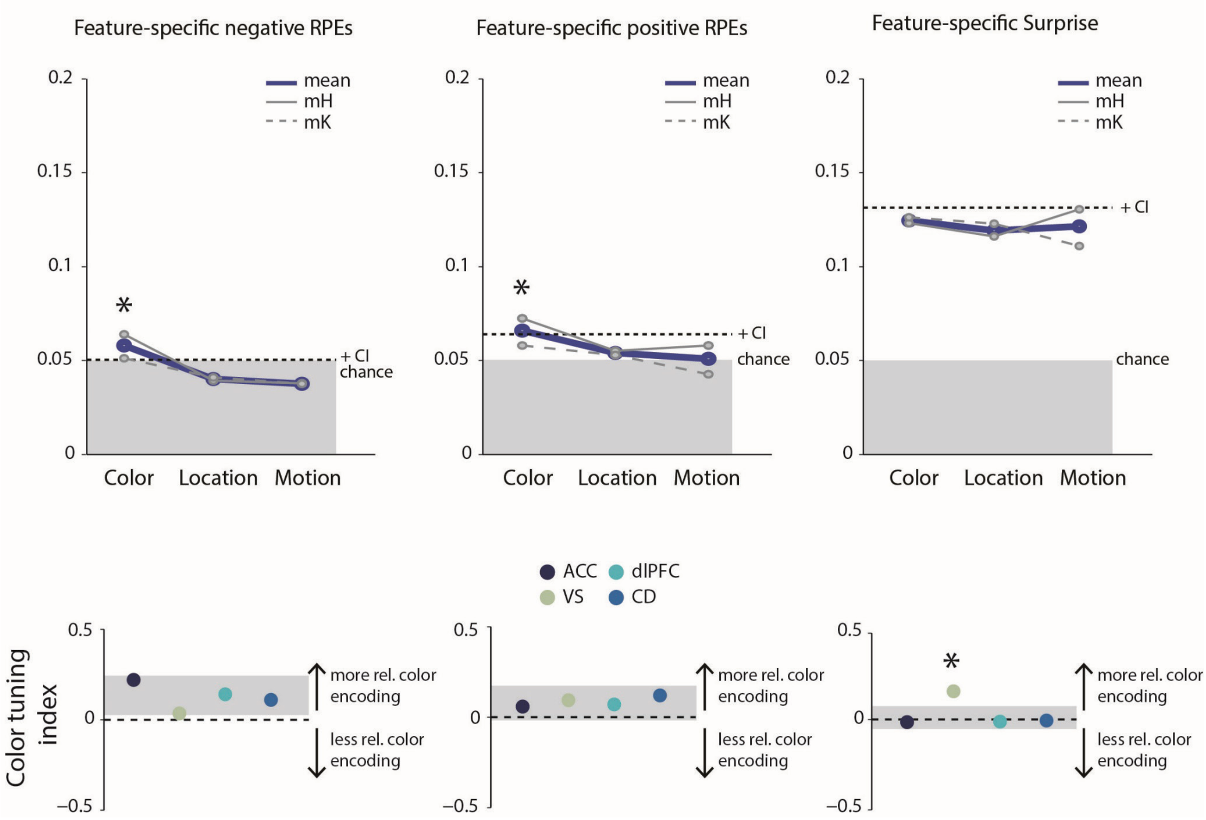

Supplementary Figure 9 | Prevalence of feature-specific negative and positive RPE.

For this analysis, we increased the threshold for identifying RPE encoding units from four consecutive bins to six consecutive bins. Top: Proportions of neurons that encode a color-, location-, or motion-specific negative, positive, or surprise RPE signal. Thick blue lines represent averages across both monkeys. Thin continuous grey lines represent data from monkey H, thin dashed grey lines represent data from monkey K. An asterisk indicates $p \leq .05$ using a one-sided bootstrap procedure that randomized the feature labels. Dotted lines indicate upper confidence interval. Grey bars indicate chance level proportion at 0.05. Bottom: Color tuning indices for each area computed according to eq. 2. Grey bar represents upper and lower bootstrap confidence interval. An asterisk indicates $p < .05$ by falling outside of the specified confidence interval. All conventions are equivalent to Figures 5, 6 of the main text.

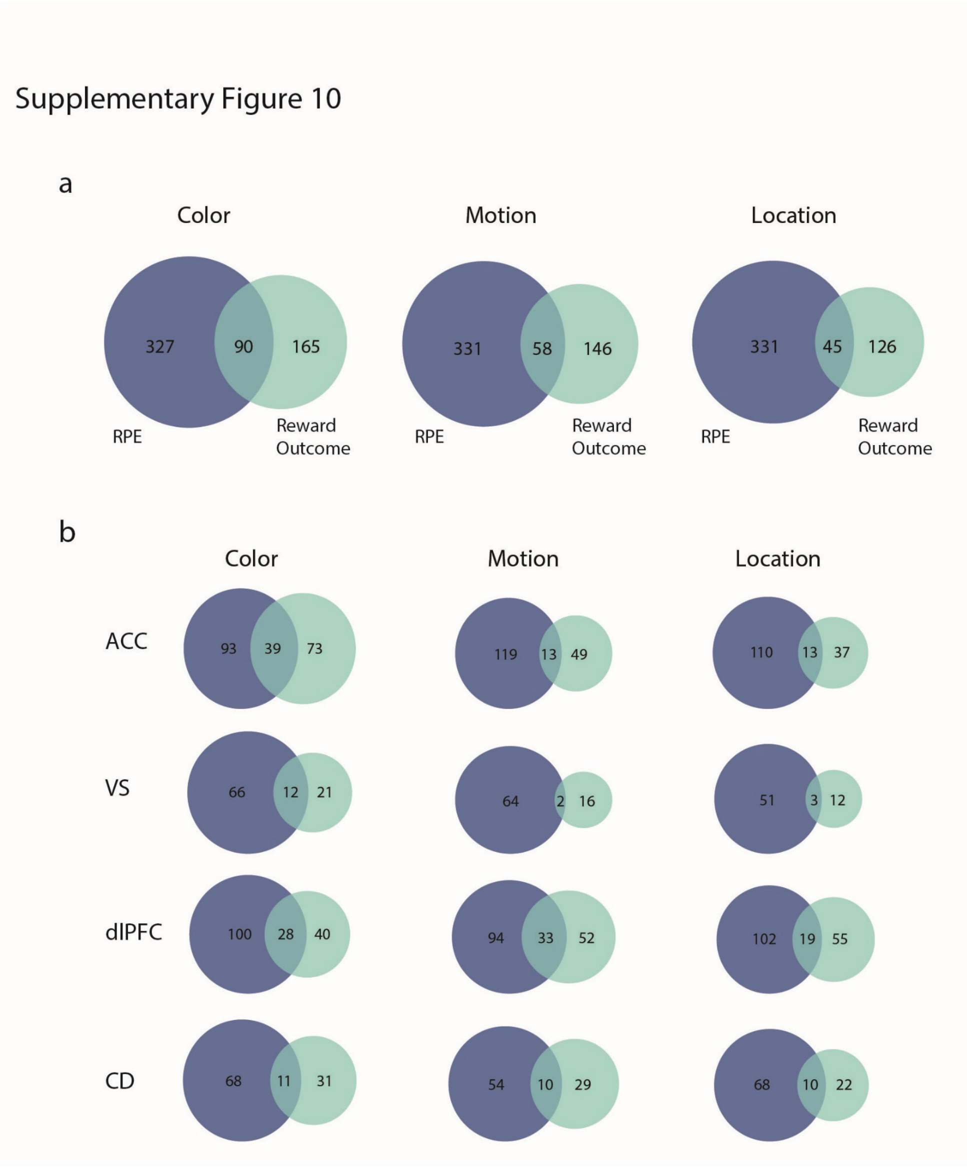

Supplementary Figure 10 | Overlap feature-specific RPE and feature-specific outcome information.

(a) Overlap of neurons encoding RPEs and reward outcome selectively for one of two colors (left), one of two motion directions (middle), or one of two locations (right). Numbers inside circles refer to numbers of neurons within that category. (b) Same conventions as for (a) split into the different areas.

Supplementary Figure 11

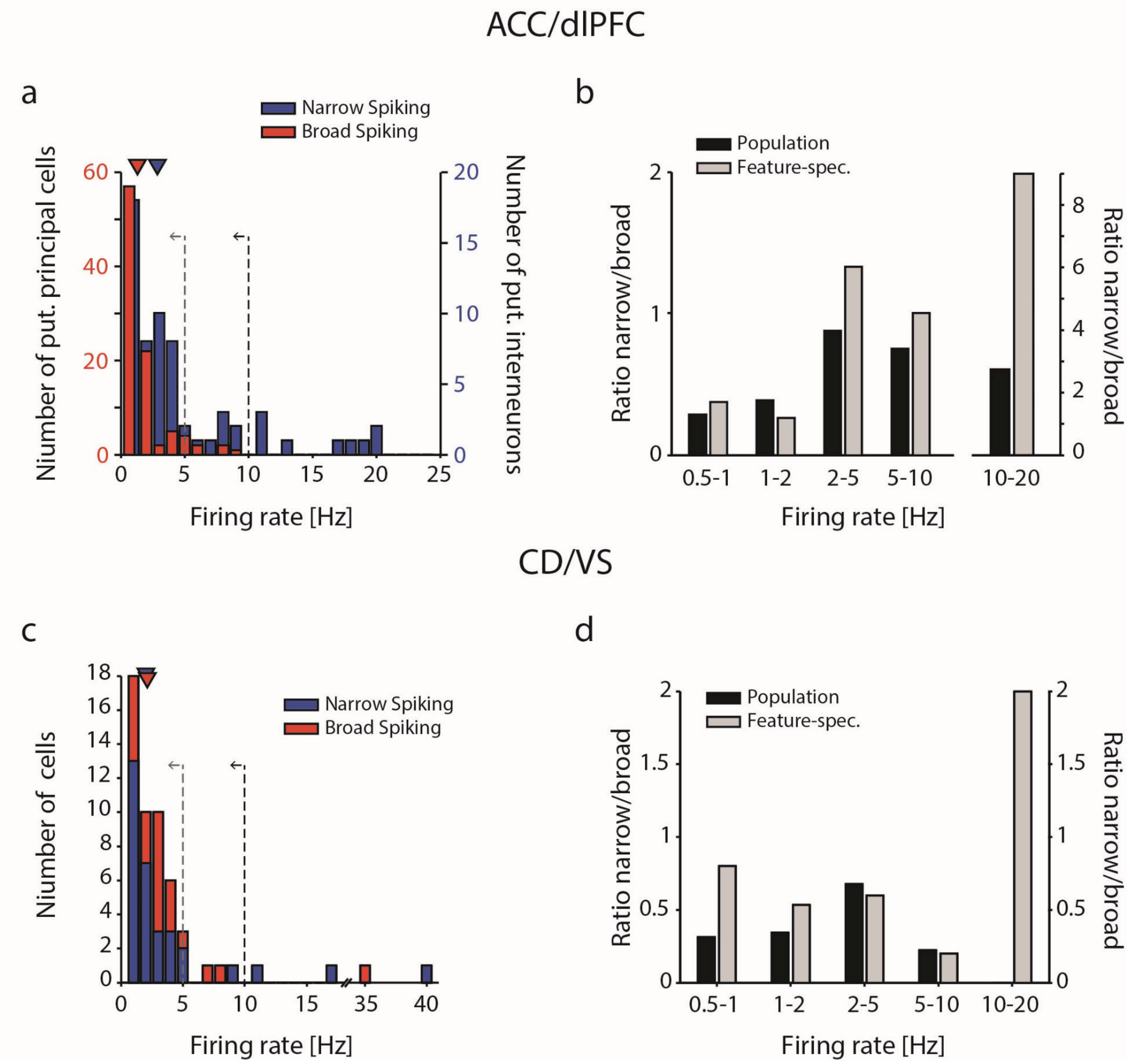

Supplementary Figure 11 | Relationship between firing rate and cell-type ratios.

(a, c) Overall distribution of firing rates for putative principal cells and putative interneurons in ACC/PFC (a) and CD/VS (c). Triangles indicate median firing rates. The greater ratio of narrow to broad spiking units for feature-specific RPE encoding units remains 1) when analysis was restricted to neurons with ≤10Hz firing rate (black dotted line) in ACC/PFC (Chi-square test, χ^2=0.11, p = 0.048) and as a trend in CD/VS (Chi-square test, χ^2=0.85, p = 0.09), and 2) when units from all regions were combined and neurons were restricted to ≤5Hz (grey dotted line) (Chi-square test, χ^2=0.66, p = 0.014). (b, d) Ratio of putative narrow to broad spiking cells in the population (black) and for feature-specific RPE encoding cells (grey) for various firing rate bins in ACC/PFC (b) and CD/VS (d). The ratio of narrow spiking to broad spiking

cells is greater for feature-specific RPE encoding neurons than for the general population in most firing rate bins.

Supplementary Figure 12

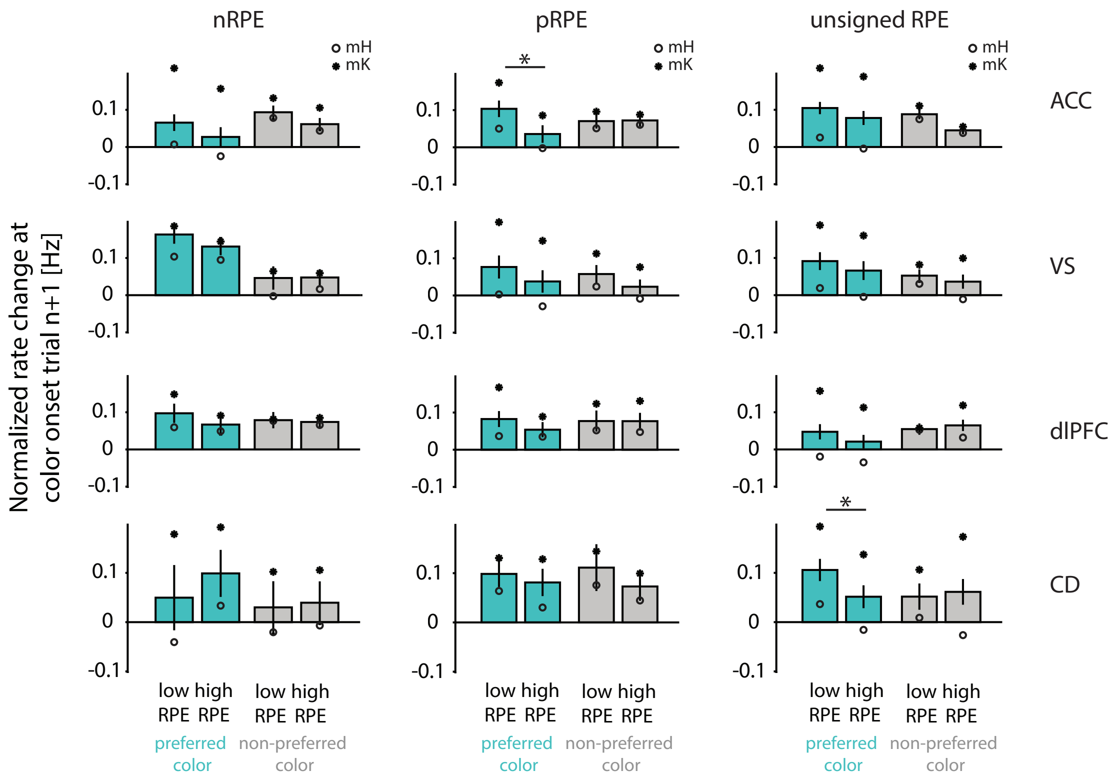

Supplementary Figure 12 | Activity at color selection following low and high RPE trials. Displayed are average normalized firing rate changes at color onset in trial n+1 across populations of color-specific RPE neurons, split by RPE type (nRPE, pRPE, unsigned RPE) and area (ACC, VS, dlPFC, CD). Rate changes were computed according to eq. 13 in Suppl. Methods and normalized to range from -1 to 1. Averages were computed separately for the 25% of trials with the greatest prediction errors and for the 25% of trials with the lowest prediction error, in cyan following preferred color choices and in grey following non-preferred color choices. Circles indicate means for monkey H, squares indicate means for monkey K. Error bars indicate SEM. Asterisks indicate significant differences in rate changes following low versus high RPEs (paired t-test, $p < .05$). Numbers of neurons included in the analysis: $n_{pRPE\text{-}ACC}=44$, $n_{pRPE\text{-}VS}=29$, $n_{pRPE\text{-}PFC}=40$, $n_{pRPE\text{-}CD}=27$; $n_{nRPE\text{-}ACC}=35$, $n_{nRPE\text{-}VS}=22$, $n_{nRPE\text{-}PFC}=40$, $n_{nRPE\text{-}CD}=17$; $n_{Surp\text{-}ACC}=87$, $n_{Surp\text{-}VS}=49$, $n_{Surp\text{-}PFC}=74$, $n_{Surp\text{-}CD}=50$.

Supplementary Figure 13

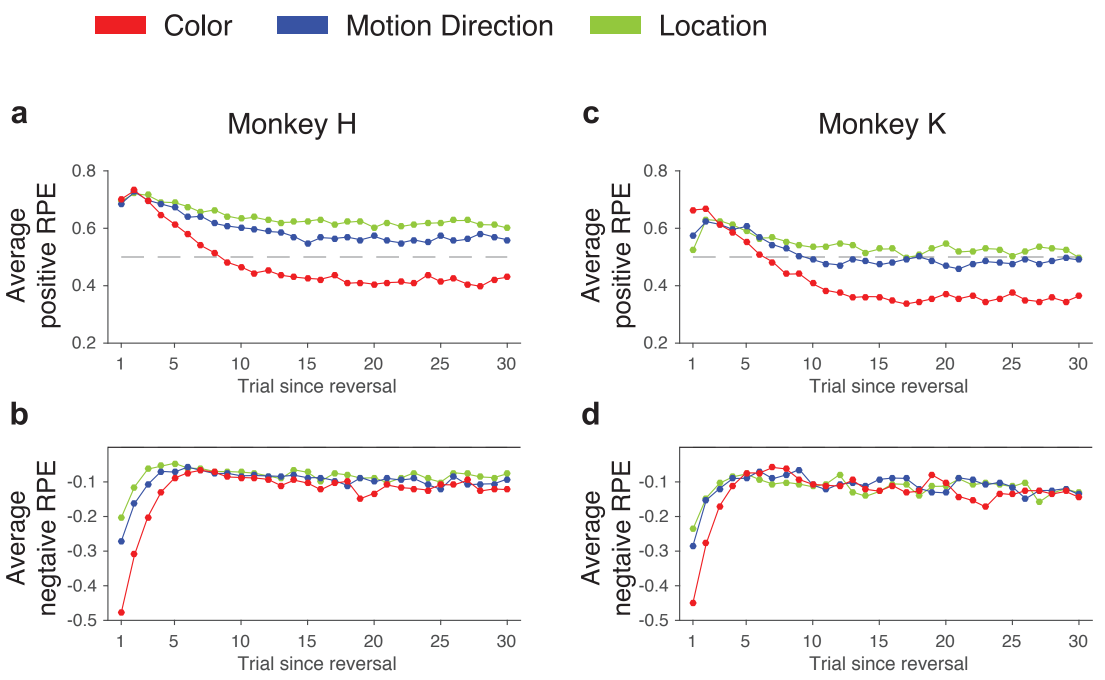

Supplementary Figure 13 | Model-derived prediction errors calculated for each stimulus. Model-derived feature-specific RPEs calculated, for visualization purposes only, separately for the three stimulus features of the chosen stimulus for positive (a, c) and negative RPEs (b, d) for monkey H (a, b) and monkey K (c, d).

Supplementary Table 1

Numbers of feature-specific RPE encoding neurons.

**Num. Neurons**	Color-specific	Location-specific	Motion-specific
Neg. RPE	114 (12.1%)	80 (8.5%)	75 (8.0%)
Pos. RPE	140 (14.9%)	106 (11.3%)	107 (11.4%)
Surprise	260 (27.6%)	258 (27.4%)	270 (28.7%)

Note that a given neuron could be entered more than once in this table if it encoded more than one feature-specific RPE. Percentages in brackets reflect out of all units considered for the analyses.

Supplementary References

1. Saleem, K. S., Kondo, H. & Price, J. L. Complementary Circuits Connecting the Orbital and Medial Prefrontal Networks with the Temporal , Insular , and Opercular Cortex in the Macaque Monkey. *J. Comp. Neurol.* **506,** 659–693 (2008).
2. Saleem, K. S., Miller, B. & Price, J. L. Subdivisions and connectional networks of the lateral prefrontal cortex in the macaque monkey. *J. Comp. Neurol.* **522,** 1641–90 (2014).
3. Smith, A. C. & Brown, E. N. Estimating a State-Space Model from Point Process Observations. *Neural Comput* **15,** 965–991 (2003).
4. Smith, A. C. *et al.* Dynamic Analysis of Learning in Behavioral Experiments. *J. Neurosci.* **24,** 447–461 (2004).
5. Hassani, S. A. *et al.* A computational psychiatry approach identifies how alpha-2A noradrenergic agonist Guanfacine affects feature-based reinforcement learning in the macaque. *Sci. Rep.* **7,** (2017).

6. Balcarras, M., Ardid, S., Kaping, D., Everling, S. & Womelsdorf, T. Attentional selection can be predicted by reinforcement learning of task-relevant stimulus features weighted by value-independent stickiness. *J. Cogn. Neurosci.* **28,** 333–349 (2016).

7. Wilson, R. C. & Niv, Y. Inferring Relevance in a Changing World. *Front. Hum. Neurosci.* **5,** 1–14 (2012).

8. Niv, Y. *et al.* Reinforcement learning in multidimensional environments relies on attention mechanisms. *J. Neurosci.* **35,** 8145–8157 (2015).

9. Ardid, S. *et al.* Mapping of functionally characterized cell classes onto canonical circuit operations in primate prefrontal cortex. *J. Neurosci.* **35,** 2975–2991 (2015).

10. Oemisch, M., Westendorff, S., Everling, S. & Womelsdorf, T. Interareal Spike-Train Correlations of Anterior Cingulate and Dorsal Prefrontal Cortex during Attention Shifts. *J. Neurosci.* **35,** 13076–89 (2015).

11. Lansink, C. S., Goltstein, P. M., Lankelma, J. V & Pennartz, C. M. A. Fast-spiking interneurons of the rat ventral striatum: temporal coordination of activity with principal cells and responsiveness to reward. *Eur. J. Neurosci.* **32,** 494–508 (2010).

12. Berke, J. D. Uncoordinated firing rate changes of striatal fast-spiking interneurons during behavioural task performance. *J. Neurosci.* **28,** 10075–10080 (2008).

13. Berke, J. D., Okatan, M., Skurski, J. & Eichenbaum, H. B. Oscillatory entrainment of striatal neurons in freely moving rats. *Neuron* **43,** 883–896 (2004).

14. Cai, X. & Padoa-Schioppa, C. Contributions of orbitofrontal and lateral prefrontal cortices to economic choice and the good-to-action transformation. *Neuron* **81,** 1140–1151 (2014).

15. Padoa-Schioppa, C. & Assad, J. A. The representation of economic value in the orbitofrontal cortex is invariant for changes of menu. *Nat. Neurosci.* **11,** 95–102 (2008).

16. Padoa-Schioppa, C. & Assad, J. A. Neurons in the orbitofrontal cortex encode economic value. *Nature* **441,** 223–226 (2006).

17. Genovesio, A., Tsujimoto, S., Navarra, G., Falcone, R. & Wise, S. P. Autonomous Encoding of Irrelevant Goals and Outcomes by Prefrontal Cortex Neurons. *J. Neurosci.* **34,** 1970–8 (2014).

18. Donahue, C. H. & Lee, D. Dynamic routing of task-relevant signals for decision making in dorsolateral prefrontal cortex. *Nat. Neurosci.* **18,** 1–9 (2015).

19. Westendorff, S., Kaping, D., Everling, S. & Womelsdorf, T. Prefrontal and anterior cingulate cortex neurons encode attentional targets even when they do not apparently bias behavior. *J. Neurophysiol.* **116,** 796–811 (2016).

20. Dunn, O. J. & Clark, V. A. Applied Statistics: Analysi of Variance and Regression. 175–178 (1987).

21. Glantz, S. & Slinker, B. Primer of Applied Regression and Analysis of Variance. 25–28 (2001).
