# Supplementary material for: Feature-specific prediction errors and surprise across macaque fronto-striatal circuits
Source: Nat Commun. 2019 Jan 11;10:176. doi: 10.1038/s41467-018-08184-9 (PMC6329800; doi:10.1038/s41467-018-08184-9)
Supplement: Supplementary file 3 — Reporting Summary [file 41467_2018_8184_MOESM3_ESM.pdf]

natureresearch

Corresponding author(s): Mariann Oemisch, Thilo Womelsdorf

Last updated by author(s): Dec 10, 2018

Reporting Summary

Nature Research wishes to improve the reproducibility of the work that we publish. This form provides structure for consistency and transparency in reporting. For further information on Nature Research policies, see Authors & Referees and the Editorial Policy Checklist.

Statistics

For all statistical analyses, confirm that the following items are present in the figure legend, table legend, main text, or Methods section.

n/a	Confirmed	
☐	☒	The exact sample size (*n*) for each experimental group/condition, given as a discrete number and unit of measurement
☐	☒	A statement on whether measurements were taken from distinct samples or whether the same sample was measured repeatedly
☐	☒	The statistical test(s) used AND whether they are one- or two-sided<br>*Only common tests should be described solely by name; describe more complex techniques in the Methods section.*
☐	☒	A description of all covariates tested
☐	☒	A description of any assumptions or corrections, such as tests of normality and adjustment for multiple comparisons
☐	☒	A full description of the statistical parameters including central tendency (e.g. means) or other basic estimates (e.g. regression coefficient) AND variation (e.g. standard deviation) or associated estimates of uncertainty (e.g. confidence intervals)
☐	☒	For null hypothesis testing, the test statistic (e.g. *F*, *t*, *r*) with confidence intervals, effect sizes, degrees of freedom and *P* value noted<br>*Give P values as exact values whenever suitable.*
☒	☐	For Bayesian analysis, information on the choice of priors and Markov chain Monte Carlo settings
☒	☐	For hierarchical and complex designs, identification of the appropriate level for tests and full reporting of outcomes
☐	☒	Estimates of effect sizes (e.g. Cohen's *d*, Pearson's *r*), indicating how they were calculated

Our web collection on statistics for biologists contains articles on many of the points above.

Software and code

Policy information about availability of computer code

Data collection	Data was collected using a multi-channel acquisition processor from Neuralynx. Neurons were sorted using the Plexon Offline Sorter. Eye position was monitored using an EyeLink SRS system. During the experiments, stimulus presentation, monitored eye positions, and reward delivery were controlled via MonkeyLogic (www.brown.edu/Research/monkeylogic/).
Data analysis	Analysis was performed with custom MATLAB code (Mathworks, Natick, MA), utilizing functions from the open-source Fieldtrip toolbox (http://www.ru.nl/fcdonders/fieldtrip/)

For manuscripts utilizing custom algorithms or software that are central to the research but not yet described in published literature, software must be made available to editors/reviewers. We strongly encourage code deposition in a community repository (e.g. GitHub). See the Nature Research guidelines for submitting code & software for further information.

Data

Policy information about availability of data

All manuscripts must include a data availability statement. This statement should provide the following information, where applicable:
- Accession codes, unique identifiers, or web links for publicly available datasets
- A list of figures that have associated raw data
- A description of any restrictions on data availability

All data supporting this study and its findings, as well as custom MATLAB code generated for analyses, are available from the corresponding author upon reasonable request.

Field-specific reporting

Please select the one below that is the best fit for your research. If you are not sure, read the appropriate sections before making your selection.

☒ Life sciences ☐ Behavioural & social sciences ☐ Ecological, evolutionary & environmental sciences

For a reference copy of the document with all sections, see nature.com/documents/nr-reporting-summary-flat.pdf

Life sciences study design

All studies must disclose on these points even when the disclosure is negative.

Sample size	Neural data was collected from two rhesus macaques, as is typical for these types of studies. We recorded a total of 1960 single neurons across four brain regions, with a range from 297-690 units per region. This sample size generally exceeds most studies reporting single neuron results.
Data exclusions	Neurons were excluded if they: 1) had a firing rate <0.5Hz within feedback epoch, 2) prediction errors could be computed for <40 trials, 3) if <40 trials could be identified as occurring either during or after learning according to an ideal observer statistic.
Replication	We sampled a large number of neurons in several brain regions in two monkeys.
Randomization	There were no experimental groups in our study.
Blinding	Blinding was not relevant to this study because there was no group allocation or drug condition etc.

Reporting for specific materials, systems and methods

We require information from authors about some types of materials, experimental systems and methods used in many studies. Here, indicate whether each material, system or method listed is relevant to your study. If you are not sure if a list item applies to your research, read the appropriate section before selecting a response.

Materials & experimental systems

n/a	Involved in the study	
☒	☐	Antibodies
☒	☐	Eukaryotic cell lines
☒	☐	Palaeontology
☐	☒	Animals and other organisms
☒	☐	Human research participants
☒	☐	Clinical data

Methods

n/a	Involved in the study	
☒	☐	ChIP-seq
☒	☐	Flow cytometry
☒	☐	MRI-based neuroimaging

Animals and other organisms

Policy information about studies involving animals; ARRIVE guidelines recommended for reporting animal research

Laboratory animals	two male rhesus macaques (macacca mulatta), age: 8 and 7 years
Wild animals	Study did not involve wild animals
Field-collected samples	Study did not involve samples collected from the field
Ethics oversight	York University Council on Animal Care

Note that full information on the approval of the study protocol must also be provided in the manuscript.
